# Supplementary material for: Detailed molecular and epigenetic characterization of the pig IPEC-J2 and chicken SL-29 cell lines
Source: iScience. 2023 Feb 20;26(3):106252. doi: 10.1016/j.isci.2023.106252 (PMC10018572; doi:10.1016/j.isci.2023.106252)
Supplement: Data S1. Complete homer output for identified motifs in Pig IPECJ-2, related to Table 2 — Homer motif analysis results for histone modifications H3K4me1, H3K4me3, H3K27ac, and enhancer elements of pig IPECJ2 cell line. P-values >1e-10 are possible false positives. Within each folder (e.g. peak_files_CTCF) are the html files showing the identified motifs when using homer (e.g. homerResults.html). [file mmc2.zip › S5/Pig_IPECJ_2/peak_files_H3K27ac/homerResults/motif42.similar.html]

motif42

## Information for motif42

T
G
A
C
T
C
G
A
C
T
G
A
T
G
C
A
C
T
A
G
A
T
G
C
C
T
A
G
T
A
C
G
T
G
A
C
A
G
T
C
G
C
A
T
G
A
T
C
A
C
G
T
  
Reverse Opposite:  

G
T
C
A
C
T
A
G
C
G
T
A
T
C
A
G
A
C
T
G
A
T
G
C
G
A
T
C
T
A
C
G
G
A
T
C
A
C
G
T
A
G
C
T
A
G
C
T
A
C
T
G
  

|  |  |
| --- | --- |
| p-value: | 1e-11 |
| log p-value: | -2.732e+01 |
| Information Content per bp: | 1.713 |
| Number of Target Sequences with motif | 28.0 |
| Percentage of Target Sequences with motif | 0.05% |
| Number of Background Sequences with motif | 5.5 |
| Percentage of Background Sequences with motif | 0.01% |
| Average Position of motif in Targets | 119.7 +/- 78.3bp |
| Average Position of motif in Background | 224.0 +/- 0.0bp |
| Strand Bias (log2 ratio + to - strand density) | -0.8 |
| Multiplicity (# of sites on avg that occur together) | 1.00 |
| Motif File: | file (matrix) reverse opposite |

### Similar de novo motifs found

|  |  |  |  |  |  |  |  |
| --- | --- | --- | --- | --- | --- | --- | --- |
| Rank | Match Score | Redundant Motif | P-value | log P-value | % of Targets | % of Background | Motif file |
